# Resveratrol retards progression of diabetic nephropathy through modulations of oxidative stress, proinflammatory cytokines, and AMP-activated protein kinase

**DOI:** 10.1186/1423-0127-18-47

**Published:** 2011-06-23

**Authors:** Chih-Chun Chang, Chieh-Yu Chang, Yang-Tzu Wu, Jiung-Pang Huang, Tzung-Hai Yen, Li-Man Hung

**Affiliations:** 1 Department and Graduate Institute of Biomedical Sciences, College of Medicine, Chang Gung University, Tao-Yuan, Taiwan; 2 Department of Nephrology and Division of Clinical Toxicology, Chang Gung Memorial Hospital, Lin-Kou Medical Center, Taipei, Taiwan

## Abstract

**Background:**

Diabetic nephropathy (DN) has been recognized as the leading cause of end-stage renal disease. Resveratrol (RSV), a polyphenolic compound, has been indicated to possess an insulin-like property in diabetes. In the present study, we aimed to investigate the renoprotective effects of RSV and delineate its underlying mechanism in early-stage DN.

**Methods:**

The protective effects of RSV on DN were evaluated in streptozotocin (STZ)-induced diabetic rats.

**Results:**

The plasma glucose, creatinine, and blood urea nitrogen were significantly elevated in STZ-induced diabetic rats. RSV treatment markedly ameliorated hyperglycemia and renal dysfunction in STZ-induced diabetic rats. The diabetes-induced superoxide anion and protein carbonyl levels were also significantly attenuated in RSV-treated diabetic kidney. The AMPK protein phosphorylation and expression levels were remarkably reduced in diabetic renal tissues. In contrast, RSV treatment significantly rescued the AMPK protein expression and phosphorylation compared to non-treated diabetic group. Additionally, hyperglycemia markedly enhanced renal production of proinflammatory cytokine IL-1β. RSV reduced IL-1β but increased TNF-α and IL-6 levels in the diabetic kidneys.

**Conclusions:**

Our findings suggest that RSV protects against oxidative stress, exhibits concurrent proinflammation and anti-inflammation, and up-regulates AMPK expression and activation, which may contribute to its beneficial effects on the early stage of DN.

## Introduction

Diabetes mellitus (DM), mainly characterized by recurrent hyperglycemia, had become one of the chronic disorders derived from insulin deficiency or resistance in the developed countries. As the high blood glucose level in diabetes persisted and progressed without appropriate medical care, relative secondary disorders involving atherosclerosis, retinopathy, nephropathy, neuropathy, stroke, and foot ulcer would individually develop with an insidious onset, which could eventually be life-threatening. Diabetic nephropathy (DN), the second most prevalent diabetes-associated complication inferior to cardiovascular disorders, impaired the renal function of DM patients and therefore cost appreciable medical labor and resource for DN management annually. Histologically featured by thickening of basement membrane, expansion and nodular aggregation of mesangial matrix (the Kimmelstiel-Wilson lesions) and sclerosis in glomeruli, DN could be multifactorial in the pathogenesis. In these risk factors, hyperglycemia was currently regarded as one of the leading causes in the progression of DN. Accumulating evidence also suggested the development of DN was associated with the activation of several stress-sensitive signal pathways, including nuclear factor kappa B (NF-κB) and mitogen-activated protein kinase (MAPK) [1-4]. Additionally, it was reported that both oxidative stress [5-8] and proinflammatory cytokines [9,10] detrimentally accelerated the pathological process of DN. Adenosine monophosphate-activated protein kinase (AMPK), a regulator of cellular energy homeostasis, was recently identified to play an important role in DN [11]. Decreased phosphorylation of AMPK was contributed to hyperglycemia-associated renal enlargement. Further studies indicated that suppression of AMPK activity was linked with oxidative stress [12] and inflammatory response [13]. Reversion of AMPK activity could ameliorate oxidative damage [14] and inflammation [15]. Thus, attention has been drawn to the modulation of AMPK signal transduction to attenuate DM-affected renal dysfunction.

Resveratrol (trans-3,4',5-trihydroxyestilbene, RSV), one naturally existing polyphenolic compound rich in grapes and several plants, was characterized as a potently free radical scavenger and antioxidative agent. Besides, RSV was pronounced to possess both cardioprotective [16-18] and antidiabetic benefits [19,20]. A vast majority of reports also supported that RSV displayed a hypoglycemic effect on DM animal models via AMPK stimulation [21-24]. In DN studies, RSV was proved to mitigate renal dysfunction and oxidative stress in type 1 diabetic rats [5,25].

One recent research investigated the AMPK-stimulating effect of RSV on the early stage of DN [26]. It was also reported that RSV did not remarkably alter the messenger RNA and protein levels of inflammation-regulatory cyclooxygenase (COX) in the diabetic rat kidneys [27]. Additionally, RSV activated NF-κB in mesangial cells under a precondition of cytokine exposure [28]. Therefore, it still required further survey to delineate the precise mechanisms of RSV action on DN.

Considering the hypoglycemic, antioxidative, inflammatory modulation and AMPK-up-regulating effects of RSV on type 1 DM, we designed this study to realize the therapeutic effects and associated mechanisms of RSV on streptozotocin (STZ)-induced DM rats. The oxidative stress, proinflammatory cytokines, and several cellular stress-activated signal pathways were simultaneously evaluated in diabetic rats. Our results show the renoprotective effects of RSV may contribute by its antioxidative and AMPK-up-regulating abilities and, to our interest, found that RSV significantly augmented inflammatory response in diabetic kidney by elevating several cytokines like tumor necrosis factor α (TNF-α) and interleukin 6 (IL-6), despite its ameliorative effect on IL-1β cytokine level in DN.

## Methods

### Animals

This survey was submitted to the rules written in the Guide for the Care and Use of Laboratory Animals, published by the US National Institutes of Health (NIH publication No. 85-23, revised 1996). Experiments were performed on male Long-Evans rats (6-7 weeks old, 220-250 g), maintained in the animal center of Chang Gung University within an environment-controlled room (ambient temperature of 25 ± 1°C and a light-dark period of 12 h) with free access to normal chow and water. The experimental animals were randomly assigned to two groups, the non-diabetic rats (control, CON) and STZ-induced diabetic rats (STZ-DM). In the diabetic group, male Long-Evans rats were fasted and anesthetized by intraperitoneal injection of pentobarbital at a dosage of 65 mg/kg. Freshly prepared STZ (65 mg/kg, Sigma-Aldrich, St. Louis, MO, USA) solution was then injected intravenously into the femoral vein of animals. The experimental rats with symptoms as polyphagia, polydipsia, and polyuria together with a blood glucose level above 300 mg/dl were considered diabetic. The blood glucose level was determined by the glucose oxidase method (chemistry analyzer; Auto Analyzer Quik-Lab, Ames, Spain). Two weeks after the onset of DM, the DM rats were further divided into three subgroups concomitantly treated with vehicle (STZ-DM), RSV 0.1 mg/kg (DM-R0.1) or RSV 1 mg/kg (DM-R1) for 7 consecutive days. RSV (Sigma-Aldrich, St. Louis, MO, USA) was suspended in 0.9% saline solution and administrated by oral gavage. At the end of RSV treatment course, the rats were euthanized and sacrificed. All the renal tissues and blood samples were preserved at -80°C.

### Western blot analysis

Tissue lysates were extracted from renal tissues in accordance to previously published procedure with appropriate modifications [16]. Briefly, dissected renal tissues were segmented into small pieces and pestled with liquid nitrogen. The grinding renal tissue samples were lysed with ice-cold lysis buffer containing 50 mM Tris-HCl (pH 7.4), 50 mM glycerophosphate, 20 mM sodium fluoride, 2 mM sodium orthovanadate, 2 mM Ethylenedinitrilotetraacetic acid (EDTA), 1 mM phenylmethanesulfonyl fluoride (PMSF), and 1% 2-mercaptoethanol. The homogenates were centrifuged at 12,000 *g *for 10 min at 4°C and the supernatants were isolated for Western blot preparation.

After protein determination, the samples were then separated by sodium dodecyl sulfate polyacrylamide gel electrophoresis (SDS-PAGE) on 10 or 15% polyacrylamide denaturing gels and thus transferred onto polyvinylidene difluoride (PVDF) membranes, which were then probed with monoclonal antibodies with recommended dilution of manganese superoxide dismutase (MnSOD), copper-zinc SOD (CuZnSOD) (Upstate Biotechnology, Lake Placid, NY, USA), NF-κB, Erk, phospho-Erk (Thr202/Tyr204), p38, phospho-p38 (Thr180/Tyr182) (Chemicon, Temecula, CA, USA), JNK, phospho-JNK (Thr183/Tyr185) (Cell Signaling Technology, Cell Signaling, Boston, MA, USA), Akt, phospho-Akt (Thr308) (Santa Cruz), AMPK and phospho-AMPK (Thr172) (Chemicon), respectively. Following the incubation with appropriate secondary horseradish peroxidase (HRP)-conjugated IgG antibodies, the chemiluminescence was thus performed. The obtained protein bands were scanned and quantified with the aid of Image J software (NIH, Bethesda, MD, USA).

### Oxidative stress and proinflammatory cytokines analysis

The renal production of superoxide anion was measured by modified lucigenin-enhanced chemiluminescence. The chemical specificity of this light-yielding reaction for superoxide anion has previously been described in detail [29]. The extent of lipid peroxidation was determined using the modified thiobarbituric acid reactive substances (TBARS) method, which was also reported previously [29]. The level of protein carbonyl group was measured by the 2,4-dinitrophenylhydrazine (DNPH) method with slight modification, as described previously [30]. Furthermore, proinflammatory cytokines in renal tissues, including TNF-α, IL-1β and IL-6, were determined by using commercially acquired ELISA kits (R&D Systems, Minneapolis, MN) according to the manufacturer's instructions.

### Histopathological analysis

To assess the renal pathology in the diabetic animal models, periodic acid-Schiff (PAS) stain was performed as described previously [31]. In brief, the kidneys were immediately perfused with 4% paraffinparaformaldehyde after the decapitation of animals. The fixed renal tissues were paraffin-embedded and cross-sectioned into 2 μm thickness and periodic acid, Schiff's reagent and hematoxylin were further performed. After dehydration, the histopathological changes of stained glomeruli were observed and elucidated by the veteran pathologist with expert guidance.

### Biological analysis

All the biological measurements were determined using commercially available kits. The blood samples from experimental animals were obtained following by overnight fasting. Plasma glucose levels were evaluated on the basis of the glucose oxidase-catalyzed reaction (chemistry analyzer; Auto Analyzer Quik-Lab, Ames, Spain). Plasma insulin levels were measured by a sandwich enzyme-linked immunosorbent assay (ELISA) method (Mercodia, Uppsala, Sweden). Plasma cholesterol, triglyceride, creatinine, and blood urea nitrogen (BUN) levels were determined under the instructions provided by the manufacturer (Ransel kit, Randox, UK).

### Statistical analysis

All values were expressed as mean ± standard error (SE). Following the performance by one-way analysis of variance (ANOVA), the difference of experimental data was analyzed by using Student *t *test. P < 0.05 was considered to be significant.

## Results

### Effects of RSV on the body weight and biochemical parameters of the STZ-DM rats

As shown in Table 1, blood glucose level was significantly higher in STZ-induced type 1 diabetic rats than in the normal controls. It was also observed that both body weight and plasma insulin levels were significantly decreased in the diabetic group when compared with the non-diabetic controls. Besides, the plasma cholesterol and triglyceride levels were significantly elevated in the diabetic rats in comparison with the non-diabetic controls. Giving RSV treatment with two dosages (0.1 and 1 mg/kg/day for 7 days) significantly ameliorated the body weight loss, hyperglycemia, hypoinsulinemia, and hyperlipidemia in STZ-DM rats, but the body weight, plasma glucose, insulin, and triglyceride levels in RSV-treated diabetic rats still remained significantly higher in comparison with normal control.

**Table 1:** The biochemical parameters of CON, STZ-DM, DM-R0.1 and DM-R1 rats

	CON (n = 11)	STZ-DM (n = 7)	DM-R0.1 (n = 11)	DM-R1 (n = 15)
Body weight (g)	420.77 ± 8.88	280.29 ± 9.41*	330.77 ± 9.45*^†^	312.81 ± 10.55*^†^
Plasma glucose (mg/dl)	137.15 ± 10.86	566.33 ± 45.24*	444.17 ± 22.90*^†^	376.48 ± 35.56*^†^
Plasma insulin (μg/l)	2.00 ± 0.28	0.11 ± 0.06*	0.78 ± 0.25*^†^	0.86 ± 0.12*^†^
Plasma cholesterol (mg/dl)	67.30 ± 9.63	111.13 ± 16.98*	59.51 ± 7.16^†^	48.05 ± 5.64^†^
Plasma triglycerides (mg/dl)	88.27 ± 11.41	166.41 ± 35.43*	55.84 ± 7.47*^†^	63.52 ± 9.20^†^
Blood urea nitrogen (mg/dl)	16.27 ± 0.94	25.01 ± 3.90*	19.97 ± 1.52*	21.73 ± 1.72*
Plasma creatinine (mg/dl)	0.37 ± 0.04	0.59 ± 0.10*	0.41 ± 0.04^†^	0.48 ± 0.04

Values were expressed as means ± standard error (n = 7~15 per group). CON: non-diabetic control, STZ-DM: streptozotocin-induced diabetes, DM-R0.1: DM treated with RSV (0.1 mg/kg/day) for 7 days, DM-R1: DM treated with RSV (1 mg/kg/day) for 7 days.

*: P < 0.05 vs. control, †: P < 0.05 vs. STZ-DM, ‡: P < 0.05 vs. DM-R0.1.

### Effects of RSV on renal function and morphology in the STZ-DM rats

**Figure 1:**
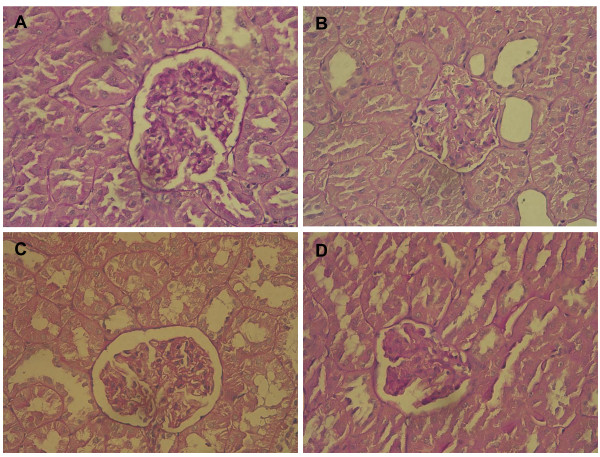
**The effects of RSV on mesangial expansion in DN**. Photomicrographs of rodent glomeruli sections of CON (A), STZ-DM (B), DM-R0.1 (C) and DM-R1 (D) groups were represented at × 400 magnification from periodic acid-Schiff-stained kidney. CON: non-diabetic control, STZ-DM: streptozotocin-induced diabetes, DM-R0.1: DM treated with RSV (0.1 mg/kg/day) for 7 days, DM-R1: DM treated with RSV (1 mg/kg/day) for 7 days.

It appeared that plasma creatinine and BUN levels were significantly increased in the diabetic rats (Table 1). Only treatment with the dosage of 0.1 mg/kg RSV markedly ameliorated the plasma creatinine level. There was no remarkable attenuation on the BUN levels after RSV treatment in the DM rats. The BUN levels in RSV-treated diabetic rats still remained significantly higher in comparison with normal control.Additionally, the renal tissue stained by Periodic acid and Schiff's solution appeared normal glomeruli in the renal cortex of non-diabetic controls (Figure 1A). In contrast, it was shown that diabetes-induced histopathological changes in the renal tissues, including the expansion of mesangial matrix and thickening of glomerular basement membrane, to a mild extent (Figure 1B). After RSV treatment, enlargement of mesangia in glomeruli was mildly attenuated in the diabetes-affected renal tissues (Figure 1C, D).

### RSV ameliorated oxidative stress in the STZ-induced type 1 diabetic kidneys

**Figure 2:**
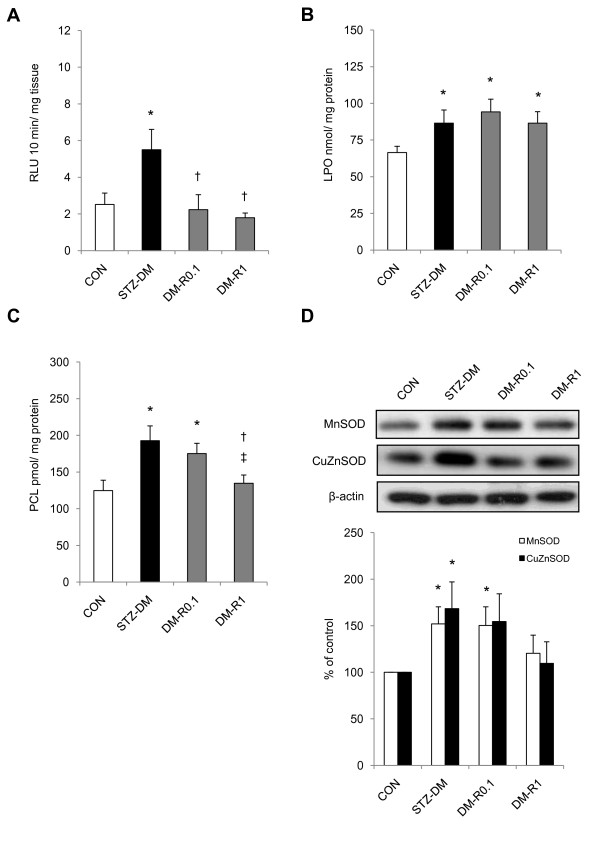
**The effects of RSV on superoxide anion production, lipid peroxidation, protein carbonyl level, MnSOD and CuZnSOD protein expressions in the renal tissues of STZ-DM rats**. (A) Superoxide anion content was measured by lucigenin-enhanced chemiluminescence. (B) Lipid peroxidation was determined using the modified thiobarbituric acid reactive substances method. (C) Protein carbonyl level was evaluated by 2,4-dinitrophenylhydrazine method. (D) Equal amounts of proteins were resolved on 10 and 15% SDS-PAGE and blotted with MnSOD and CuZnSOD antibodies, respectively. The blots were shown at the top and the quantified ratios were shown at the bottom. Results were expressed as means ± standard error (n = 5 per group). *: P < 0.05 vs. control, †: P < 0.05 vs. STZ-DM, ‡: P < 0.05 vs. DM-R0.1, RLU: relative light unit, LPO: lipid peroxidation, PCL: protein carbonyl level, SOD: superoxide dismutase, CON: non-diabetic control, STZ-DM: streptozotocin-induced diabetes, DM-R0.1: DM treated with RSV (0.1 mg/kg/day) for 7 days, DM-R1: DM treated with RSV (1 mg/kg/day) for 7 days.

The indicators of oxidative stress including the contents of superoxide anion, malondialdehyde, and carbonyl protein, and protein expressions of MnSOD and CuZnSOD were all significantly enhanced in the nephritic tissues of STZ-DM rats than that in the normal control (Figure 2). After RSV administration, the superoxide anion and protein carbonyl levels were significantly decreased in the diabetic rats (Figure 2A, C). Additionally, it seemed that RSV did not alleviate lipid peroxidation when compared to the non-treated DM rats (Figure 2B). Lipid peroxidation was significantly increased in RSV-treated diabetic groups when compared to normal control. Although MnSOD and CuZnSOD showed a reductive tendency in a dose-dependent manner after RSV treatment, there was no statistical significance (Figure 2D). MnSOD protein expression in DM-R0.1 group was still increased in comparison with normal control.

### RSV significantly decreased IL-1β cytokine level but enhanced TNF-α and IL-6 contents in the diabetic kidney without the involvement of NF-κB signaling pathway

**Figure 3:**
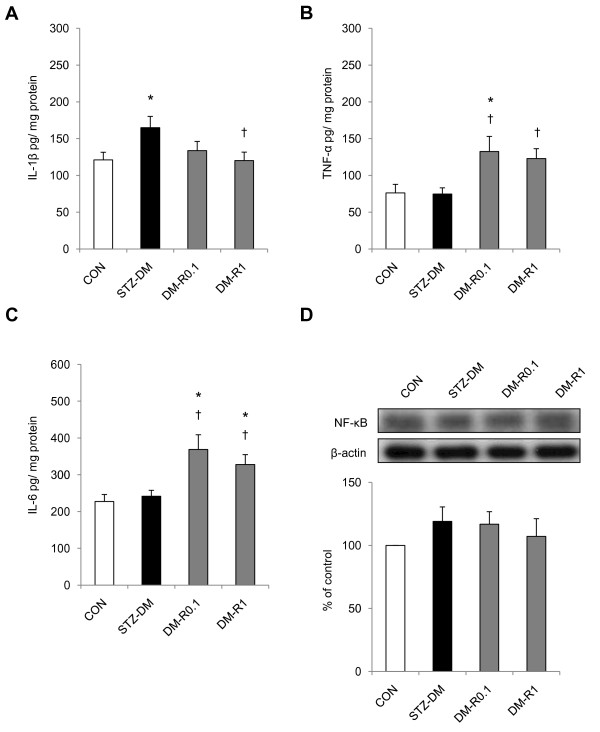
**The effects of RSV on IL-1β, TNF-α, IL-6, and NF-κB protein expression in the renal tissues of STZ-DM rats**. Samples were processed to measure IL-1β (A), TNF-α (B), or IL-6 (C) using a competitive ELISA. (D) Equal amounts of proteins were resolved on 10% SDS-PAGE and blotted with NF-κB antibody. The blots were shown at the top and the quantified ratios were shown at the bottom. Results were expressed as means ± standard error (n = 5 per group). *: P < 0.05 vs. control, †: P < 0.05 vs. STZ-DM, ‡: P < 0.05 vs. DM-R0.1, IL-1β: interleukin 1β, TNF-α: tumor necrosis factor α, IL-6: interleukin 6, NF-κB: nuclear factor kappa B, CON: non-diabetic control, STZ-DM: streptozotocin-induced diabetes, DM-R0.1: DM treated with RSV (0.1 mg/kg/day) for 7 days, DM-R1: DM treated with RSV (1 mg/kg/day) for 7 days.

It was observed the content of proinflammatory cytokine IL-1β significantly increased in the diabetic kidneys in comparison with the normal controls (Figure 3A). There was no significant elevation in renal TNF-α and IL-6 cytokine levels in the STZ-DM rats when compared with the control group (Figure 3B, C). RSV treatment significantly reduced IL-1β levels in the kidney of DM-R1 rats when compared to that of the STZ-DM group. In the diabetic kidneys, however, RSV treatment remarkably enhanced the proinflammatory cytokine TNF-α and IL-6 expressions. Renal cytokine TNF-α and IL-6 levels were also significantly higher in RSV-treated groups when compared to normal control. It seemed the NF-κB p65 subunit was not contributed to the cytokine expressions because neither the STZ-induced diabetic nor the RSV-treated DM rats revealed significant differences when compared with the non-diabetic control (Figure 3D).

### The antidiabetic effect of RSV on renal tissues had no remarkable association with MAPK signaling pathway

**Figure 4:**
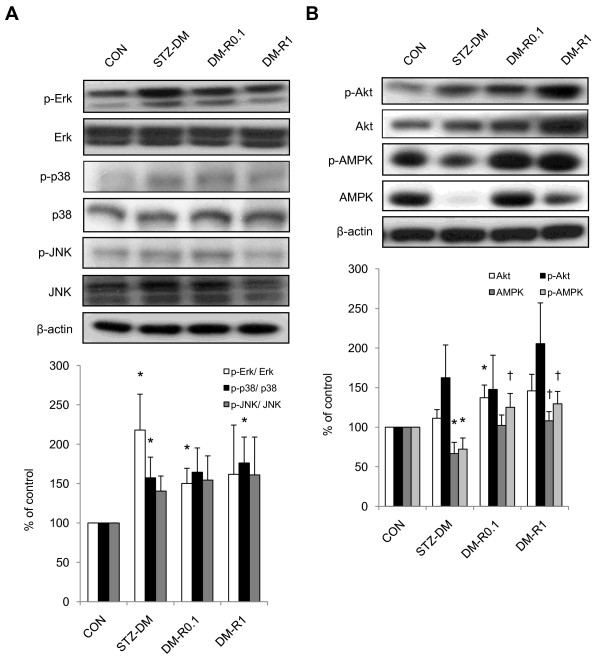
**The effects of RSV on Erk, p38, JNK, Akt, and AMPK phosphorylation and total protein expressions in the renal tissues of STZ-DM rats**. Equal amounts of proteins were resolved on 10% SDS-PAGE and blotted with Erk, p-Thr202/Tyr204-Erk, p38, p-Thr180/Tyr182-p38, JNK, p-Thr183/Tyr185-JNK (A), and Akt, p-Thr308-Akt, AMPK, p-Thr172-AMPK (B) antibodies. The blots were shown at the top and the quantified ratios were shown at the bottom. Results were expressed as means ± standard error (n = 5 per group). *: P < 0.05 vs. control, †: P < 0.05 vs. STZ-DM, ‡: P < 0.05 vs. DM-R0.1, CON: non-diabetic control, STZ-DM: streptozotocin-induced diabetes, DM-R0.1: DM treated with RSV (0.1 mg/kg/day) for 7 days, DM-R1: DM treated with RSV (1 mg/kg/day) for 7 days.

The diabetic induction significantly increased both expressions of phosphorylated Erk and p38 proteins (Figure 4A). Although no statistical significance was shown, there was an augmentative tendency in JNK activation in the diabetic kidneys when compared to the non-diabetic controls. RSV administration in the diabetic group did not significantly lower the ratio of Erk phosphorylation, albeit a decreasing tendency was shown. There was also no remarkable influence on p38 and JNK phosphorylations in the renal tissues of the RSV-treated diabetic group in comparison with that of STZ-DM rats. The ratio of Erk phosphorylation was significantly elevated in DM-R0.1 group when compared to normal control, and that of p38 phosphorylation was significantly increased in DM-R1 group in comparison with normal control.

### RSV significantly attenuated AMPK signal reduction, with less attribution to Akt expression, in STZ-induced type 1 diabetic kidneys

A significant reduction in total and phosphorylated forms of AMPK expressions was observed in the kidneys of STZ-DM rats (Figure 4B). It was revealed that RSV treatment significantly increased AMPK phosphorylation and protein expression in the diabetic kidneys. Though there was no statistical significance, it was shown that an increased tendency of Akt phosphorylation and protein expression in the diabetic kidneys and RSV treatment augmented this tendency. In addition, the renal expression of phosphorylated Akt was also slightly elevated without significance in the STZ-DM group. Akt protein expression was significantly increased in RSV-treated diabetic rats when compared to normal control.

## Discussion

In the present study, we claimed that RSV significantly prevented loss of body weight, lowered plasma glucose and creatinine concentrations, and increased plasma insulin level, to moderate extents in the STZ-diabetic rats. Additionally, RSV remarkably alleviated oxidative stress and prevented AMPK protein down-regulation may contribute to its renoprotective effects in the diabetic rats. Interestingly, our experimental results further revealed that RSV significantly reduced the levels of IL-1β but elevated that of TNF-α and IL-6 in the diabetic kidney. To our knowledge, this is the first report to investigate the concurrently suppressive and stimulating effects of RSV on proinflammatory cytokines in the renal tissues of diabetes in vivo.

It has been shown that hyperglycemia promoted oxidative stress in nephritic tissues, eventually leading to renal injury in diabetes. Augmentation of free radicals and impairment of key antioxidant enzymes were believed to contribute to the development of DN. The ameliorative effects of RSV on hyperglycemia-associated oxidative stress were widely recognized [25]. RSV was proved to possess an insulin-like property in vivo [19]. Further, there was increasing evidence implicating that RSV alleviated oxidative stress in a variety of hyperglycemia-affected tissues, including renal [25], neuron [32], vascular endothelial [33], and pancreatic β cells [34]. One recent study indicated that RSV prevented lipid peroxidation and increased glutathione contents and activities of SOD and catalase in STZ-induced diabetic kidneys [25]. It was also reported that RSV decreased the generation of reactive oxygen species (ROS) and nitric oxide in high glucose-exposed porcine renal proximal tubular cells [35]. However, our results suggested that RSV partially attenuated hyperglycemia-associated oxidative injury mediated by reduction of superoxide anion and protein carbonyl levels, but did not alleviate lipid peroxidation in renal tissues.

Attraction has been drawn to the correlation between inflammatory activity and diabetic complications. There was accumulating evidence indicating that renal inflammation played a key role in the pathogenesis of DN. it was demonstrated that diabetes increased proinflammatory cytokines including TNF-α, IL-1β and IL-6 in the circulating [36], renal production [37,38], and urinary excretion [39]. Our results revealed excessive renal production of IL-1β in the early stage of DN. Administration of RSV significantly inhibited IL-1β elevation in the diabetic kidneys. RSV treatment, however, elevated TNF-α and IL-6 levels in the renal tissues of the diabetic group. Recently, several researches have proved that RSV did not suppress but augmented signals responsible for inflammation in the renal tissues of diabetes. Gene and protein expressions of COX, one prostaglandin synthase mainly activated in certain inflammatory conditions, were not suppressed by RSV treatment in the renal tissues of diabetic rodents [27]. Instead, RSV enhanced NF-κB activity in the renal tubular and mesangial cells exposed to cytokine mixtures [28]. Although these reports indicated the proinflammatory potential of RSV which was similar to our experimental results, they were contrastive to previous studies identifying the anti-inflammatory property of RSV in diabetes. We showed that RSV modulated proinfammatory cytokines but unaffected NF-κB signals, implying a possibility that RSV modulates inflammation mediated by other existing cascades against cellular stress in DN. The effects of RSV on renal inflammation in DM remain to be further elucidated.

Under physiological circumstances, the signaling regulations of cellular energy like insulin cascades were predominated by both Akt and AMPK. It was demonstrated that suppression of AMPK was interposed by hyperglycemia and elevated Akt activity [11]. In addition, a recent study revealed that reduced phosphorylation of AMPK appeared to be reversed under RSV treatment in the diabetic kidney [26]. Our finding suggested that RSV prevented renal AMPK dephosphorylation and protein down-regulation in insulin-deficient diabetic rats. It was observed a tendency of increased Akt phosphorylation in the diabetic kidney, without any remarkable influence after RSV treatment. Since AMPK was newly identified as a modulating factor in diabetes-induced renal injury, RSV treatment may play a novel role as a therapeutic agent by prevention of AMPK dephosphorylation and protein down-regulation in early-stage DN.

## Conclusion

In conclusion, the present study provides evidence that RSV reduced plasma glucose and creatinine, oxidative stress, proinflammatory cytokines and up-regulated AMPK proteins in diabetes which may contribute to its renoprotective effects in the early stage of DN. Interestingly, RSV decreased proinflammatory cytokine IL-1β but elevated TNF-α and IL-6 levels in renal tissues of STZ-induced diabetic rats. If and how RSV influences renal inflammation still remains controversial. RSV also prevented AMPK protein dephosphorylation and down-regulation in the insulin-deficient diabetic kidney. These findings suggest that RSV may serve as one useful new therapeutic agent in the early stage of DN.

## Competing interests

The authors declare that they have no competing interests.

## Authors' contributions

CCC and LMH designed research. CCC performed experiments. CYC, YTW, JPH, and THY helped CCC in experiments. CCC and LMH analyzed the data. CCC and LMH wrote the paper. All authors read and approved the final manuscript.
